# Stochastic epigenetic outliers can define field defects in cancer

**DOI:** 10.1186/s12859-016-1056-z

**Published:** 2016-04-22

**Authors:** Andrew E. Teschendorff, Allison Jones, Martin Widschwendter

**Affiliations:** CAS Key Lab of Computational Biology, CAS-MPG Partner Institute for Computational Biology, Shanghai Institute for Biological Sciences, Chinese Academy of Sciences, Shanghai, China; Statistical Cancer Genomics, Paul O’Gorman Building, UCL Cancer Institute, University College London, 72 Huntley Street, London, WC1E 6BT UK; Department of Women’s Cancer, University College London, 74 Huntley Street, London, WC1E 6AU UK

**Keywords:** DNA methylation, Field defect, Cancer, EWAS, Differential variability, Differential methylation, Stochastic

## Abstract

**Background:**

There is growing evidence that DNA methylation alterations may contribute to carcinogenesis. Recent data also suggest that DNA methylation field defects in normal pre-neoplastic tissue represent infrequent stochastic “outlier” events. This presents a statistical challenge for standard feature selection algorithms, which assume frequent alterations in a disease phenotype. Although differential variability has emerged as a novel feature selection paradigm for the discovery of outliers, a growing concern is that these could result from technical confounders, in principle thus favouring algorithms which are robust to outliers.

**Results:**

Here we evaluate five differential variability algorithms in over 700 DNA methylomes, including two of the largest cohorts profiling precursor cancer lesions, and demonstrate that most of the novel proposed algorithms lack the sensitivity to detect epigenetic field defects at genome-wide significance. In contrast, algorithms which recognise heterogeneous outlier DNA methylation patterns are able to identify many sites in pre-neoplastic lesions, which display progression in invasive cancer. Thus, we show that many DNA methylation outliers are not technical artefacts, but define epigenetic field defects which are selected for during cancer progression.

**Conclusions:**

Given that cancer studies aiming to find epigenetic field defects are likely to be limited by sample size, adopting the novel feature selection paradigm advocated here will be critical to increase assay sensitivity.

**Electronic supplementary material:**

The online version of this article (doi:10.1186/s12859-016-1056-z) contains supplementary material, which is available to authorized users.

## Background

Feature selection presents an important statistical challenge in the analysis of omic data [1–3]. It is most often encountered in the context of supervised analyses where one wishes to find features that are informative of differences between two phenotypes of interest (POI). The standard paradigm is to identify features for which the average level of the molecular mark of interest (e.g. DNA methylation or gene expression) is significantly different between two POI, using well-known tests such as Student’s t-test, its regularized/moderated versions [4–6], or non-parametric equivalents such as the Wilcoxon rank sum (or Mann–Whitney) test [7]. However, an often overlooked problem when applying t-tests, or their non-parametric equivalents, to omic data, is that these tests are underpowered to detect biological outliers, i.e. infrequent (heterogeneous) changes of considerable magnitude, which occur mainly, if not exclusively, within one phenotype. A number of recent studies have highlighted the potential importance of such heterogeneous, stochastic, outlier events in disease aetiology [8–15]. For instance, one study measured DNA methylation in precursor cervical cancer samples and showed that DNA methylation outliers in these cytologically normal lesions were predictive of progression to neoplasia [13]. It is therefore entirely plausible that such DNA methylation outliers may, in general, define epigenetic cancer field defects [16–18], i.e. molecular alterations in normal cells which later undergo neoplastic transformation.

Given that t-tests and other standard statistical tests are unsuitable for identifying epigenetic outliers, we proposed a novel feature selection paradigm based on the concept of differential variability (DV) [13, 14]. A growing concern however is that outliers could in principle also reflect genetic and technical factors [19]. Because of this, a number of DV tests have emerged, with improved statistical properties, notably with an improved control of the type-1 error rate [19–21]. As pointed out by these studies, if differentially variable outliers were technical artefacts, then certain DV tests such as Bartlett’s test (or F-test) would suffer from a very high type-1 error rate. On the other hand, given that cancer studies profiling precursor lesions are generally limited by sample size, DV tests which exhibit good control of the type-1 error rate, may also be seriously underpowered to detect the biologically interesting outliers.

Henceforth, we here conduct a detailed comparison of five different DV algorithms on a total of five DNA methylation data sets, encompassing over 700 samples, including two of the largest studies profiling precursor cancer lesions [13, 22]. We demonstrate marked variation in the ability of DV algorithms to identify true positives, with deep and far-reaching implications for studies seeking to identify epigenetic field defects in cancer and possibly also in other complex diseases.

## Methods

### DNA methylation datasets

We analysed a total of 5 DNA methylation data sets (see Additional file 1: Table S1 for summary).

#### Precursor and cancer DNA methylation datasets

Our primary DNA methylation data sets focused on the profiling of precursor cancer lesions and are available from the GEO website (www.ncbi.nlm.nih.gov/geo/) under accession numbers GSE30758 and GSE69914.

Dataset GSE30758 consists of 152 cytologically normal cervical smear samples, representing prospectively collected samples within the ARTISTIC trial, with 75 of the women who provided a sample developing a cervical intraepithelial neoplasia of grade 2 or higher (CIN2+) three years after sample collection [13]. In order to test whether CpGs identified from GSE30758, i.e. CpGs that correlate with the risk of CIN2+, show more progressive changes in CIN2+ and cervical cancer we used three other data sets (GSE20080, GSE37020, GSE30759) profiling normal cervical and CIN2+ or cervical cancer samples (Additional file 1: Table S1). All of these datasets were generated using Illumina Infinium 27 k beadarrays and we used the normalized data, as described by us previously [13, 14].

Dataset GSE69914 was generated using Illumina Infinium 450 k beadarrays and consists of 50 normal breast tissue samples from healthy women, a set of 42 matched normal-adjacent breast cancer pairs (a total of 84 samples), and a further 263 unmatched breast cancers. Raw data was processed with *minfi* [23] using the *preprocessRaw* function, the Illumina definition for methylation signal in *getBeta* and estimating P-values of detection with *detectionP* using total intensity “m + u”. Type-2 probe bias was corrected using BMIQ [24]. Subsequently, we tested for batch effects by performing a SVD on the intra-sample normalized data matrix, and checking which factors (biological or technical) the top components of variation were correlating with. The top components of variation in this data matrix correlated with biological factors, notably normal-cancer status.

### Statistical algorithms for differential variability (DV)

We compared a total of 5 algorithms/statistical tests, aimed at identifying differentially variable features. The five DV algorithms/tests are (i) Bartlett’s test [25], (ii) a novel DV algorithm, which we call “iEVORA” (similar to the original EVORA-Epigenetic Variable Outliers for Risk prediction Analysis algorithm [13, 14] ), (iii) a joint test for differential means and differential variance in DNA methylation (“J-DMDV”) [20], (iv) an empirical Bayes Levene-type test (“DiffVar”) [19] and (v) a test based on a generalized additive model for location and scale (“GAMLSS”) [21]. With the exception of iEVORA, which we present here for the first time, all other DV algorithms (i.e. BT/EVORA, J-DMDV, DiffVar, GAMLSS) have been previously used in cancer epigenome or EWAS studies [13, 21, 26].

#### BT & iEVORA

Briefly, Bartlett’s test (BT) is similar to an F-test for testing homoscedasticity, and is well-known to be sensitive to single outliers. Because of this, we also consider a regularized version of it, which we call iEVORA, whereby features deemed significant by Bartlett’s test are re-ranked according to an ordinary differential methylation statistic (e.g. the statistic from a t-test). To clarify this further, *P*-values from Bartlett’s test are used to estimate corresponding false discovery rate (FDR) values using the Q-value method [27] and a threshold (typically FDR < 0.05) used to select significantly DV features. These significantly DV features are then re-ranked according to their differential methylation statistic. Thus, in iEVORA, significance is assessed at the level of differential variability, using Bartlett’s test, but significant DV features with larger changes in the average DNA methylation are favored over those with smaller shifts in average DNA methylation. This re-ranking strategy therefore ensures that DV features driven by single, or a few, outliers are only ranked highly if there are no features which are differentially methylated in terms of mean DNAm levels.

#### J-DMDV, DiffVar and GAMLSS

The third algorithm (“J-DMDV”), proposed by Wang and Ahn [20], works in the M-value (M = log_2_[β/(1-β)] basis and uses a joint score test for mean and variance within a linear regression framework. *P*-values from this test are converted into Q-values (FDR) and features selected (and also ranked) according to a FDR < 0.05 threshold. The fourth algorithm (“DiffVar”) is based on an empirical Bayes extension of the Levene-test [19]. Briefly, this algorithm first computes the square (or absolute) deviations of samples within a phenotype from the corresponding group (phenotype) mean using the M-value basis. It then uses the framework of moderated t-tests [5], to compare the distribution of deviations between the two phenotypes. *P*-values from this test are converted into Q-values (FDR) and features selected (and also ranked) according to a FDR < 0.05 threshold. The final algorithm (“GAMLSS”) was developed by Wahl et al. [21] within the GAMLSS (Generalized Additive Models for Location, Scale and Shape) framework. This algorithm also works in the M-value basis, and here we adapt it to run on 3 separate generalized linear additive models within a nested framework: a null model without mean and variance, a regression model for the mean only and a model for the mean and variance. Two likelihood ratio tests are then constructed by comparing the log-likelihoods of the mean-only model to the null, and the mean + variance model to the mean-only model. This yields two *P*-values for each feature, and features are deemed significant if at least one of these two *P*-values is less than a nominal threshold (after adjustment for multiple testing using a FDR < 0.05 threshold). Thus, GAMLSS will yield significant hits if there are differences in terms of mean DNAm. We also note that our implementation of GAMLSS does not compare a variance-only model to the null, since the algorithm aims to identify additional features where variance “adds predictive value” over a model which only includes the mean.

### Software availability (iEVORA)

The iEVORA algorithm is freely available as an executable R-script, and can be found as a Supplementary Software file as part of the accompanying publication, see [22].

### Evaluation of DV algorithms to detect true DVCs on simulated data

In order to compare the DV algorithms to each other, we devised a simulation framework allowing also for different types of differential variability. In each simulation run we generated an artificial DNA methylation data matrix consisting of 6000 CpGs and 100 samples. Samples were subdivided into two phenotypes, a “normal” and a “disease state”, each comprising 50 samples. We declared 600 CpGs to be truly differentially variable, allowing for 3 different types of DV, with 200 CpGs in each type. The remaining 5400 CpGs are not differentially variable. These are modelled from a beta-value distribution *B(a1,b1)* with *a1 = 10* and *b1 = 90,* i.e. we assume that these CpGs are generally unmethylated with a mean beta value of 0.1, with a standard deviation of approximately +/− 0.03. For the 600 true positives, a proportion of the samples in the “disease” phenotype are modelled from a beta-value distribution *B(a2,b2)* with *a2 = 6* and *b2 = 4,* i.e. a distribution with mean value 0.6 and a standard deviation of approximately +/− 0.15. We note that although in this simulation we consider all CpGs to be unmethylated in the normal state, that there is no loss of generality, since mathematically, there is a complete symmetry between unmethylated and methylated CpGs. Thus, for the 600 true DVCs and for a number of samples in the disease phenotype, there will be an average increase in DNAm of ~0.5. The 600 true DVCs however fall into 3 categories of DV. For 200 of these CpGs, we model all samples in the disease phenotype from *B(a2,b2).* Thus, these DVCs will typically also differ in terms of the mean level of DNA methylation and in fact, will exhibit stronger differences in terms of the mean DNAm than in terms of differential variance. Hence, these 200 DVCs are of “type-1a” DV. For another 200 CpGs, we only allow 20 of the 50 disease phenotype samples to be modelled from *B(a2,b2)*, with rest of the samples being modelled from *B(a1,b1).* Thus, for these DVCs, half of the disease samples exhibit increases in DNAm, with the rest being indistinguishable from the normal phenotype. For these CpGs, differential variance is the key discriminatory characteristic, although they will still exhibit significant differences in terms of mean DNAm since a reasonable fraction of the disease samples exhibit deviations from the normal state. These DVCs are of “type-1b” differential variability. Finally, for the last set of 200 true positives, we only allow 3 disease samples to differ from the normal state. For these DVCs, there is therefore no significant difference in terms of the average DNA methylation between the two phenotypes. However, the variance will differ owing to the outliers in the disease phenotype. These DVCs are defined as being of “type-2”.

We performed a total of 100 Monte Carlo runs, in each run recording 5 performance measures for each of the five DV algorithms: (1) the overall sensitivity of the DV algorithm using a FDR (false discovery rate) corrected threshold of 0.05, defined as the fraction of true DVCs identified by the DV algorithm, (2) the true FDR at the estimated FDR < 0.05 threshold where the FDR estimate was obtained using Q-values [27], (3–5) the sensitivities to detect type-1a, type-1b and type-2 differentially variable CpGs. We focused on the FDR and not the FPR (false positive rate), since it is the FDR which gives us the confidence level that a given positive is a true positive, i.e. the FDR is related to the positive predictive value (PPV) through the relation FDR = 1-PPV.

### Evaluation of DV algorithms on real DNA methylation data

Initially, we compared the algorithms in their ability to detect DVCs between normal samples from healthy women and normal samples from women who developed neoplasia or who had cancer (see section on DNAm data sets for details), without considering the likelihood of these DVCs being true positives. Thus, for each of the DV algorithms and each CpG site, we estimated *P*-values, and from these, Q-values (FDR) [27]. In the case of BT and iEVORA, *P*-values came from Bartlett’s test. In the case of DiffVar and J-DMDV, both tests provide *P*-values, as described in the respective publications. Features were deemed significant if Q < 0.05. In the case of GAMLSS, we obtained two *P*-values, one assessing whether the mean is associated with the phenotype, and another assessing whether the variance adds predictive value over the mean. Both sets of *P*-values were transformed into Q-values and features with at least one of these Q-values being less than 0.05, were selected and deemed statistically significant.

To enable a more formal comparison of the DV algorithms, we devised a strategy that would allow us to estimate the positive predictive value (PPV) of the test. The key insight or hypothesis is that DVCs obtained in the discovery set are more likely to be biological true positives if they exhibit progressive changes in DNA methylation in either neoplastic or invasive cancer tissue. A feature detected in pre-neoplastic lesions (i.e. in the discovery set) that is biological relevant is more likely to mark cells which become neoplastic and therefore one would expect enrichment of these marks in neoplasia and invasive cancer. This means that if a given “true positive” CpG site exhibits higher DNAm levels (maybe only marginally so) in precursor cancer lesions, that this same site will undergo larger and more frequent DNAm changes in cells that are neoplastic or invasive. Thus, the PPV refers to the fraction of CpGs identified in the discovery set (i.e. by comparing normal cells at risk of neoplastic transformation to normal cells that are not at risk), which exhibit progression in the independent data sets representing the more advanced stage in cancer development. Statistically, this “progression” can be measured using t-statistics from a t-test, since the t-statistic is proportional to the average deviation in DNAm from the normal state. A similar argument can be applied to the case of CpG sites that undergo marginal hypomethylation in precursor cancer lesions.

In the context of cervical carcinogenesis, we thus applied the DV algorithms to identify DVCs hypervariable in the 75 normal samples which 3 years later progressed to CIN2+ status compared to the 77 normal samples from women who remained healthy (the “ART” data set in Additional file 1: Table S1). The DNAm data for this set were generated on Illumina 27 k beadarrays, and so, because of the design of the 27 k array, we only focused on DVCs which exhibited increases in DNAm in the precursor lesions. We considered the top ranked 500, 1000 and 5000 DVCs (irrespective of FDR values attaining statistical significance). In the case of GAMLSS, which provides two *P*-values per feature, we ranked the selected features according to the significance of the DV statistic. For each DV algorithm, we then computed and compared t-statistics of these top ranked DVCs in two independent Illumina 27 k data sets profiling normal and CIN2+ samples, and another 27 k dataset of normal cervix and cervical cancers (“CIN2 + (A)&(B)” and “CC” in Additional file 1: Table S1). The fraction of DVCs attaining t-statistics larger than 1.96 (*P* < 0.05) and preserving the same directionality of change in the independent data was used as the PPV estimate. We note that although using a *P* < 0.05 in the independent data set does not correct for multiple-testing of 500, 1000 or 5000 features, that in this context it is more important to control the false negative rate (FNR). In other words, using an overly stringent significance threshold in the independent data would lead to an unnecessarily large FNR. Moreover, the same criterion is used for each method.

In the context of breast carcinogenesis, we applied the DV algorithms to identify DVCs hypervariable in the 42 normal-adjacent samples compared to the 50 normal samples from healthy women. Because of the design of the 450 k array, we now considered DVCs which exhibited either increases or decreases in DNAm in the normal-adjacent samples. We considered the top ranked 500, 1000 and 5000 DVCs in each category (irrespective of FDR values attaining statistical significance). In each case, we then computed t-statistics of these top ranked CpGs, as derived from comparing the 50 normal breast tissue samples to 305 breast cancers. The fraction of DVCs attaining t-statistics larger (lower) than 1.96 ( −1.96) (*P* < 0.05) and preserving the same directionality of change in the independent data, was used as the PPV estimate. As before, this threshold ensures a reasonable compromise between the FDR and FNR.

## Results

### DV algorithms that have strong control for the type-1 error rate do not identify differentially variable CpGs (DVCs) in studies profiling precursor cancer lesions

**Fig. 1:**
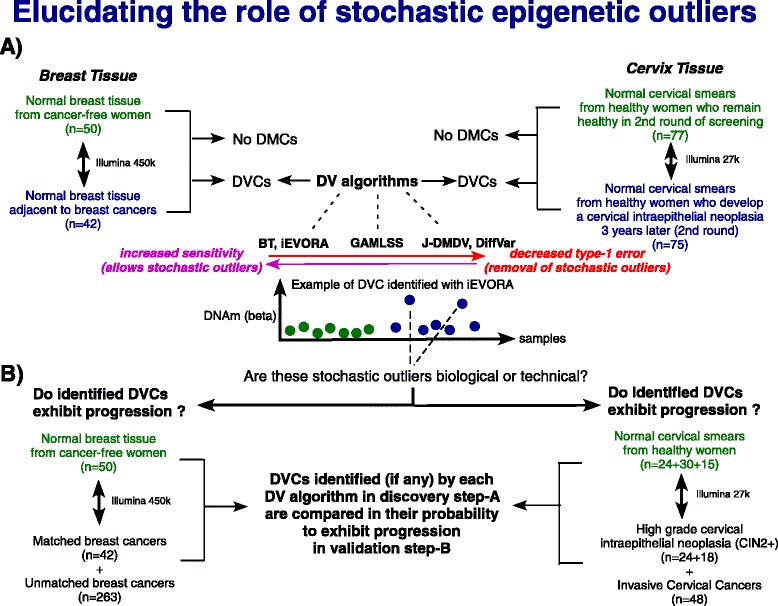
Overall strategy for comparing DV algorithms in their ability to identify field defects in cancer: **a** In a discovery step, we apply five DV algorithms (BT, iEVORA, GAMLSS, J-DMDV, DiffVar) to identify differentially variable CpGs (DVCs) between two normal phenotypes, in two tissue types, as shown. The DV algorithms differ in their sensitivity and control of type-1 error rate, with some DV algorithms not identifying stochastic outlier profiles (i.e. DNA methylation profiles with a few outliers), whilst others are sensitive to them. **b** In the validation step, we assess the identified DVCs (if any) of each DV algorithm in terms of whether they exhibit progression/enrichment within established neoplastic cells or invasive cancers. This allows an objective comparison of the DV algorithms and helps assess whether stochastic epigenetic outliers identified in step-A using algorithms such as iEVORA are biological or not

Our overall strategy is summarized in Fig. 1. Briefly, we select two DNA methylation data sets comparing normal cells from healthy individuals to normal cells which either become neoplastic a few years later (Cervix-study), or which were collected adjacent to a breast cancer (Breast-study). We compare DV algorithms in their ability to identify DVCs between these two normal phenotypes (Fig. 1a). To assess whether the DVCs are biological, i.e. whether they define putative field defects, we ask if these DVCs exhibit progression/enrichment within established neoplastic tissues (Fig. 1b). This strategy allows us to compare DV algorithms in their ability to detect field defects.

We decided to compare a total of 5 DV algorithms, with four of these having been proposed recently: (i) Bartlett’s test (BT) [13], (ii) a joint test for differential means and differential variance in DNA methylation (“J-DMDV”) [20], (iii) an empirical Bayes Levene-type test (“DiffVar”) [19] and (iv) a test based on a generalized additive model for location and scale (“GAMLSS”) [21]. As previously shown, Bartlett’s test is highly sensitive to single outliers [19–21], and assuming that single outliers are not of biological interest, this translates into a poor control of the type-1 error rate. Thus, the recently proposed J-DMDV, GAMLSS and DiffVar algorithms offer improved control of the type-1 error rate [19–21]. In addition to these 4 tests, we here devised a novel DV algorithm, which we call “iEVORA” (Methods), similar to the original EVORA (Epigenetic Variable Outliers for Risk prediction Analysis) algorithm [13, 14], and which can be thought of as providing a regularized version of Bartlett’s test.

**Fig. 2:**
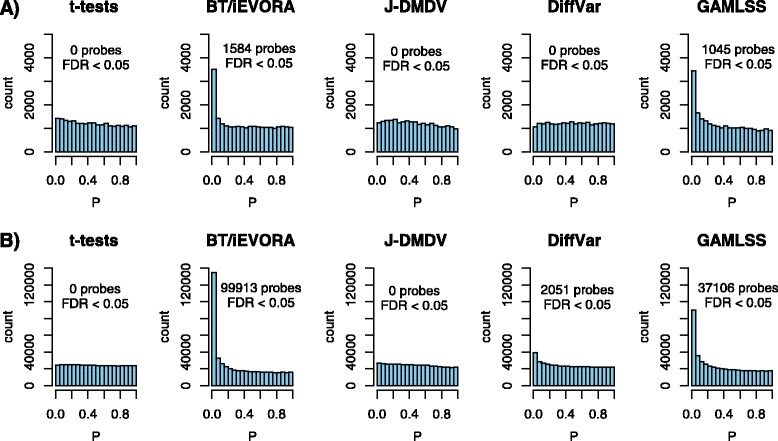
Sensitivity to detect field defects in cancer: **a** Histograms of *P*-values of five feature selection methods (as indicated) in the ARTISTIC data set comparing 75 cytologically normal samples which do not progress to CIN2+ and 77 cytologically normal cells which do progress to CIN2+ within 3 years. **b** As (**a**) but now for the PRE-BC data set comparing 50 normal breast samples from healthy women to 42 normal-adjacent samples from age-matched breast cancer patients

We first applied each of these five DV algorithms, as well as moderated t-tests, to a data set (“ARTISTIC”) which had profiled 152 cytologically normal cervical smear samples with Illumina 27 k DNA methylation beadarrays, with 75 of these 152 samples being from women who three years after sample collection developed a high grade cervical intraepithelial neoplasia (CIN2+) [13]. We note that there were no genome-wide significant differentially methylated CpGs (DMCs) between the normal samples from women who remained disease-free and the normal samples which progressed to CIN2+, as assessed using moderated t-tests (Table 1, Fig. 2a), in agreement with our previous observation [13]. We next compared all five DV algorithms in their ability to detect differentially variable CpGs (DVCs) between the same two phenotypes. We observed marked differences, with J-DMDV and DiffVar not identifying any DVCs at genome-wide significance, in stark contrast to iEVORA and GAMLSS which could identify many DVCs (Table 1, Fig. 2a). On the other hand, if we compared normal to CIN2+ samples, or normal samples to cervical cancer, we observed many DMCs and all DV algorithms had enough sensitivity to identify DVCs (Table 1).

**Table 1:** Comparison of Differential Variability (DV) and t-test feature selection algorithms on DNAm data

Feature selection algorithm	CIN2+ risk (27 k)	CIN2+ (27 k)	CC (27 k)	NADJ (450 k)	BC (450 k)
Moderated t-tests	0	2456 (10 %)	13009 (50 %)	0	345479 (71 %)
Bartlett-test (BT)	**1584 (7 %)**	3475 (15 %)	17846 (69 %)	**99913 (21 %)**	400689 (82 %)
IEVORA	**1584 (7 %)**	3475 (15 %)	17846 (69 %)	**99913 (21 %)**	400689 (82 %)
DiffVar	0	202 (<1 %)	8928 (35 %)	2051 (<1 %)	268027 (55 %)
J-DMDV	0	1973 (8 %)	11632 (45 %)	0	416995 (86 %)
GAMLSS2	1045 (4 %)	3263 (14 %)	16626 (64 %)	37106 (<1 %)	434657 (89 %)

The rows label the name of the feature selection algorithm, the number of identified features associated with different phenotypes at an FDR < 0.05. The phenotypes considered are prospective risk of CIN2+ (i.e. precursor CIN2+ lesions, *n* = 75), CIN2+ (cervical intraepithelial neoplasia of grade 2 or higher, *n* = 24), CC (cervical cancer, *n* = 48), normal breast tissue adjacent to a breast cancer (NADJ, *n* = 42), and breast cancer (BC,*n* = 305). In the context of the cervix, the reference phenotype were normal cervical samples profiled in each study (*n* = 77, 24 and 15, respectively). In the context of breast, the reference were 50 normal breast tissue samples from healthy women. We note that since Bartlett’s-test and IEVORA only differ in the ranking order of significant features, that their values here are identical. In boldface we indicate the algorithm(s) identifying most DVCs in each of the two normal-to-normal comparisons

The algorithms performed similarly in a second data set, measuring DNA methylation (now Illumina 450 k beadarrays) in over 300 samples, including 50 normal breast tissue samples from healthy women, 42 normal-adjacent breast tumor matched pairs and an additional 263 unmatched breast cancers (Methods). In this independent set we could also not detect any DMCs between the normal cells from healthy women and the normal cells adjacent to breast cancers (Table 1, Fig. 2b). The two DV algorithms which in the cervical smear analysis could not identify any DVCs, could also not identify any DVCs in this set (J-DMDV), or in the case of DiffVar, not as many as GALMSS or iEVORA (Table 1, Fig. 2b). In agreement with the cervical study, if we compared the normal samples from healthy women to breast cancers, we observed that most sites in the genome constituted DMCs, as well as DVCs, and that any DV algorithm could identify DVCs (Table 1).

### DVCs pinpoint epigenetic field defects which progress to invasive cancer

**Fig. 3:**
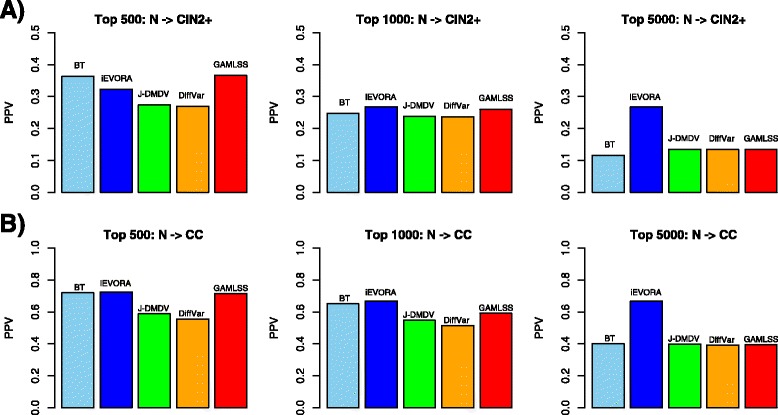
Positive Predictive Values (PPVs) of DVCs identified from pre-neoplastic lesions in cervical neoplasia and invasive cervical cancer. **a** PPVs of differentially variable CpGs (DVCs) selected by each of five different DV algorithms from the ARTISTIC data set (comparing 75 normal cervical smear samples from women who 3 years later developed a CIN2+ to 77 from women who remained disease free), with the PPV values estimated in an independent Illumina 27 k set profiling 24 normal cervical smears (N) and 24 CIN2+ samples. The number of top-ranked selected DVCs increases along the panels from left to right. The PPV was estimated as the fraction of hypermethylated DVCs attaining a t-statistic larger than 1.96 (*P* < 0.05) in the independent set. Only hypermethylation was considered due to the design of the 27 k beadarray which is overrepresented for probes in gene promoters. **b** As (**a**), but now for an independent Illumina 27 k set profiling 15 normal cervical tissue (N) and 48 invasive cervical cancers (CC)

The increased sensitivity of iEVORA and GAMLSS to detect DVCs in pre-neoplastic lesions does not necessarily mean that these DVCs are biological features of relevance to the carcinogenic process. However, if DVCs detected between normal and pre-neoplastic lesions exhibit progressive changes in neoplasia and invasive cancer, then this would support their biological relevance. Thus, we compared all the algorithms in their ability to detect CpG sites in pre-neoplastic lesions, which later progress in neoplasia and/or invasive cancer (Methods). In the context of cervical carcinogenesis, progression was assessed using two independent data sets profiling normal and CIN2+ samples, as well as a data set profiling normals and invasive cervical cancers [14]. We observed that DVCs selected and ranked using iEVORA, Bartlett’s test (BT) or GAMLSS were more likely to undergo further significant DNAm changes (preserving directionality) in CIN2+ and cervical cancer compared to features selected using t-tests, or one of the other DV algorithms (J-DMDV and DiffVar) (Fig. 3, Additional file 1: Figures S1-S2). iEVORA was more robust than BT and GAMLSS, attaining positive predictive values (PPV) for CIN2+ of over 25 % and for cervical cancer of over 60 % across a larger range of top ranked DVCs (Fig. 3).

**Fig. 4:**
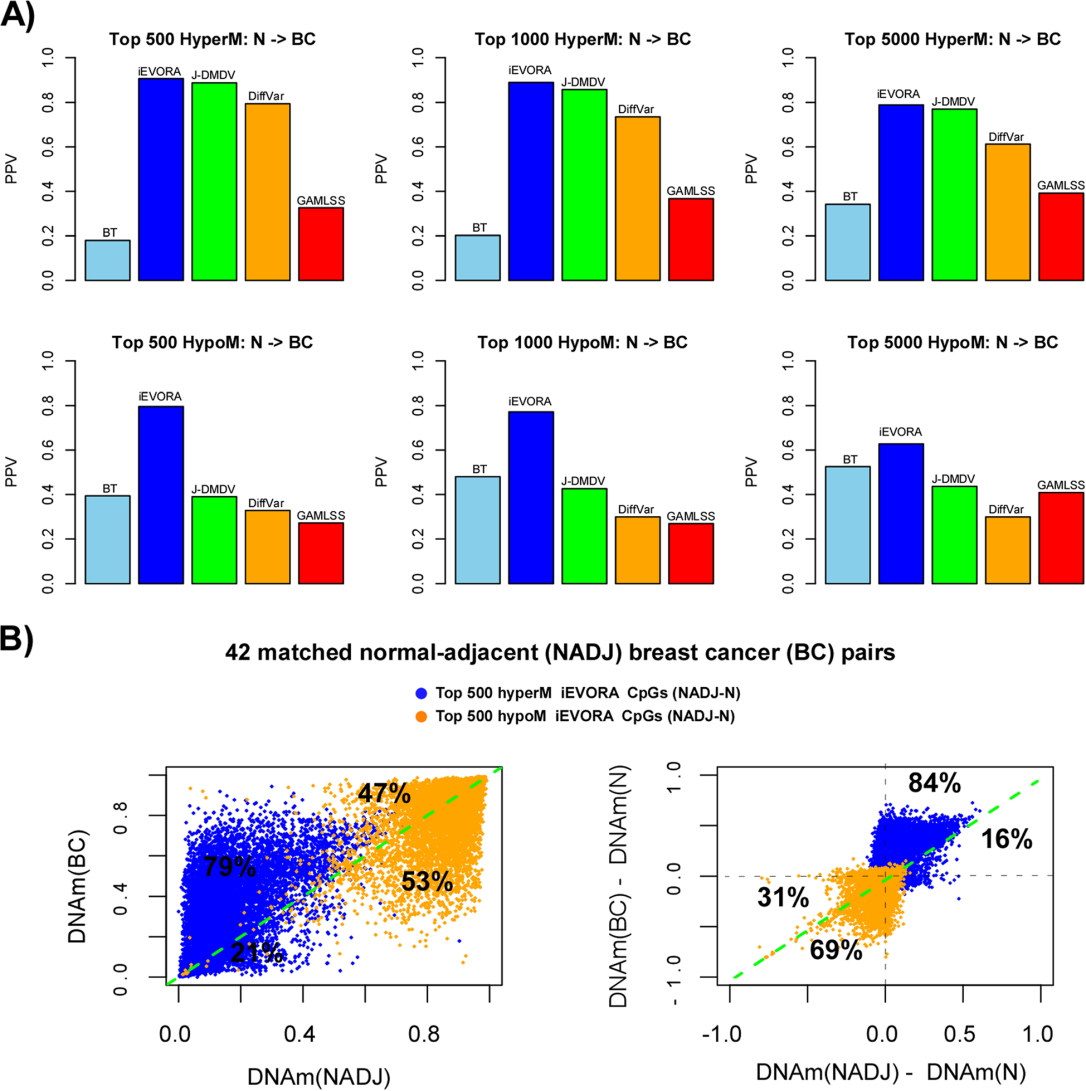
Positive Predictive Values (PPVs) and progression of DVCs from normal-adjacent tissue in invasive breast cancer. **a** PPVs of differentially variable CpGs (DVCs) selected by each of five different DV algorithms from comparing 50 normal breast samples from cancer-free women to 42 normal samples adjacent to breast cancers, with the PPVs estimated in 306 invasive breast cancers (compared to same 50 normal reference samples). The number of top-ranked selected DVCs increases along the panels from left to right. PPVs were estimated for hyper-and-hypomethylated DVCs separately: in the case of hypermethylated (hypomethylated) DVCs, PPV was estimated as the fraction of these CpGs attaining a t-statistic larger (lower) than 1.96 (*P* < 0.05) when comparing invasive cancer to normal. **b** Left panel: for the top 500 DVCs selected using IEVORA (comparing normal breast samples to normal-adjacent breast tissue), scatterplots compare the DNA methylation values of these sites in the 42 normal adjacent samples (x-axis, NADJ) to the corresponding DNA methylation values in the matched breast cancers (y-axis, BC). Observe how hypermethylated DVCs tend to exhibit further increases in DNAm in the breast cancers that are matched to their corresponding normal-adjacent tissue, whereas the opposite is true for hypomethylated DVCs. Right panel: as left panel, but now plotting the difference in DNAm between the normal-adjacent sample and normals (x-axis,NADJ-N) to the corresponding difference in DNAm between the matched breast cancer and normals (BC-N). We note that because each data point corresponds to 1 CpG site in one patient who provided a normal-adjacent and breast cancer sample, that some of the hypermethylated (hypomethylated) DVCs may exhibit lower (higher) methylation in some of the normal-adjacent samples compared to the normal state

iEVORA also outperformed all other DV algorithms in the context of breast carcinogenesis, where progression was assessed by comparing the 50 normal breast samples from healthy women to the 305 breast cancers. In most cases, iEVORA achieved PPVs for breast cancer of around 80 % or over, in stark contrast to BT or GAMLSS, whose PPVs never exceeded 40 % (Fig. 4a, Additional file 1: Figure S3).

To further demonstrate the biological relevance of DVCs in breast cancer progression, we selected the top ranked 500 DVCs (using iEVORA) between the 50 normal breast samples and the 42 normals adjacent to breast cancers and further compared their DNA methylation values in the 42 matched breast cancers. This showed that approximately 80 to 86 % of the DVCs which were hypermethylated in normal-adjacent tissue (compared to normals from healthy women), exhibited additional DNAm increases in the matched breast cancers (Fig. 4b). A similar pattern was observed for the case of DVCs that were hypomethylated in normal-adjacent tissue, although the association was less striking (Fig. 4b). Together, these results demonstrate that iEVORA is able to identify epigenetic field defects which exhibit further progressive changes in breast cancer.

### Types of DV in DNA methylation and their dynamics in carcinogenesis

**Fig. 5:**
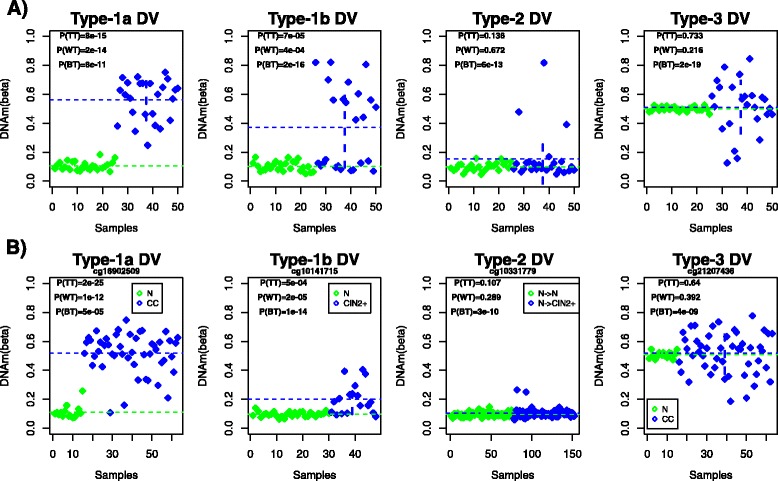
Types of differential variability. **a** Simulated examples of different types of DV arising in DNAm studies, with y-axis labelling the DNAm fraction. The horizontal dashed lines indicate the mean in each phenotype. Phenotype is labelled by a different color. *P*-values from a t-test (TT), a Wilcoxon rank sum test (WT) and Bartlett’s test (BT) are given. Bartlett’s test is a test for differential variance. **b** Real data examples of the types of DV shown in (**a**). The horizontal dashed lines indicate the mean in each phenotype. Phenotype is labelled by a different color. N = normal, CIN2 + =cervical intraepithelial neoplasia of grade 2 or higher (non-invasive), CC = cervical cancer (invasive). *P*-values from a t-test (TT), a Wilcoxon rank sum test (WT) and Bartlett’s test (BT) are given. Bartlett’s test is a test for differential variance

Next, we sought to understand why there are such marked differences among DV algorithms to detect DVCs in the earliest stages of carcinogenesis, whilst differences are less marked in later stages (Table 1). Based on extensive data analysis of DNA methylation datasets [13], we first categorized DV into 3 broad classes (Fig. 5, Methods). We define “type-1 DVCs” as those which also differ significantly in terms of average DNA methylation levels (Fig. 5a), with a further subdivision into “type-1a” and “type-1b” depending on whether the DVCs exhibit stronger differences in the mean or variance (Fig. 5a). In contrast to type-1 DVCs, type-2 and type-3 DVCs only show differences at the level of DNAm variance, with average levels of DNAm in each phenotype being statistically indistinguishable (Fig. 5a). The key difference between type-2 and type-3 DVCs is that in the type-2 case, the increased variance is driven by few outliers exhibiting coordinated changes (i.e. in the same direction), whereas in the type-3 case, the increased variance is potentially due to more outliers but with a larger level of discoordination, with outliers exhibiting both hyper and hypomethylation (Fig. 5a). Real data examples confirm the existence of these different types of DV (Fig. 5b), and although most of these focus on hypermethylation, analogous types of DV exhibiting hypomethylation are also observed (Additional file 1: Figure S4).

**Fig. 6:**
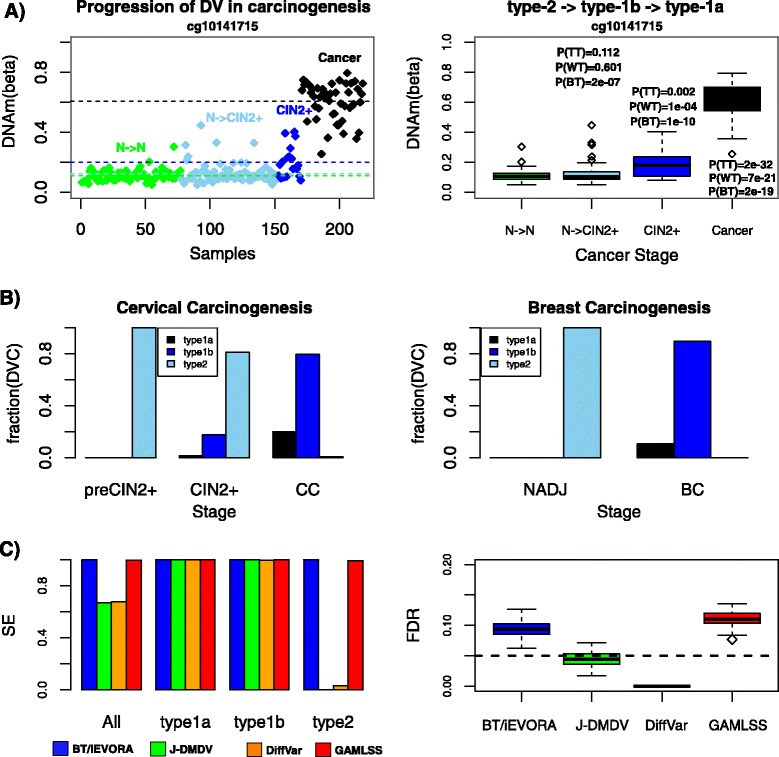
The dynamics of DV in carcinogenesis and operating characteristics of DV algorithms. **a** Progression of DV in cervical carcinogenesis. *Left panel* depicts the DNAm beta-value of a specific CpG (cg10141715) across different disease stages in cervical carcinogenesis, including cytologically normal cells which remain normal 3 years later (N- > N), cytologically normal cells which progress to CIN2+ 3 years later (N- > CIN2+), cervical intraepithelial neoplasia of grade 2 or higher (CIN2+) and cervical cancer. *Right panel* is a boxplot representation, indicating the *P*-values from a t-test (TT), Wilcoxon rank sum test (WT) and Bartlett’s test (BT) between the normal state (N- > N) and each of the other 3 stages. **b** Relative fractions of type-1a, type-1b and type-2 DVCs in cervical and breast carcinogenesis. DVCs were selected using an FDR threshold of 0.05 on the Bartlett’s test *P*-value. They were defined to be of type-2 if the t-test *P*-value was not significant (*P* > 0.05). They were defined to be of type-1 if the t-test *P*-value < 0.05, and of type-1a if the t-test *P*-value was more significant than the one from the Bartlett’s test, otherwise defined as type1-b. In the context of the cervix, the reference samples were normal cervical samples from the corresponding study. In the context of breast, the reference samples were normal breast tissue samples from healthy women. **c** Left panel: Barplots of estimated sensitivity (SE) values averaged over 100 simulated runs for a number of different DV algorithms (standard deviations were small and not shown for convenience). DVCs were selected at an estimated FDR < 0.05. Shown are the overall sensitivities to detect any DVC, and the corresponding sensitivities to detect particular types of DV. Right panel: Boxplots of the true FDRs for each DV algorithm. Green dashed line indicates the line FDR = 0.05

Demonstrating that this taxonomy of DV is of biological relevance, we observed that DVCs typically exhibited progressive changes in DNA methylation in carcinogenesis, evolving from being type-2 DVCs in the earliest stages of cancer to being type-1 DVCs in neoplasia (Fig. 6a, Additional file 1: Figure S5). Confirming this dynamics of DV on a global scale, we observed that type-1 and type-2 DV exhibited widely different frequencies depending on disease stage, with type-1 DV being very infrequent in pre-neoplastic lesions but much more prominent in neoplasia and invasive cancer (Fig. 6b).

Thus, we posited that the variable performance of DV algorithms and the critical dependence on disease stage, could be explained by their varying sensitivities to detect different types of DV. To this end, we conducted a simulation study, where we simulated DVCs from the type-1a, type-1b and type-2 subtypes, and then compared the sensitivity of the different algorithms to detect them (Methods). We observed that Bartlett’s test, iEVORA and GAMLSS were able to retrieve all types of true DVCs with equal power without losing much control of the false discovery rate (FDR) (Fig. 6c). In contrast, although J-DMDV and DiffVar could achieve much better control of the FDR, their power to detect type-2 DV was clearly compromised (Fig. 6c).

### Outlier DVCs are not markers of immune or stromal cells, but are enriched for transcription factor binding sites and PRC2/bivalent target genes

In principle, one could argue that outlier DVCs are the result of alterations in tissue composition caused by changes in stromal or immune-cell infiltrates. If so, we reasoned that a Gene Set Enrichment Analysis (GSEA) would reveal enrichment of biological terms related to stromal or immune cell-types [28–30]. Performing a GSEA using an expanded Molecular Signatures Database (MSigDB) [31], which included sets of transcription factor binding sites, as implemented by us previously [22, 32], we did not observe however any evidence for enrichment of stromal or immune-cell type terms among hypervariable DVCs (Additional file 2). In fact, hypervariable DVCs only exhibited a strong enrichment for bivalently marked genes and binding sites of transcription factors specifying chromatin architecture, including PRC2, RAD21 and CTCF (Additional file 1: Table S2 and Additional file 2: Table S2). Thus, together, these results support the view that changes in the stromal/immune cell composition of the normal tissues are not driving the specific outlier DVCs as identified using iEVORA.

### Outlier DVCs are underenriched for cross-reactive and polymorphic probes

If outlier DVCs are biological features, marking cancer field defects, we reasoned that these DVCs would also be underenriched for probes that have been deemed to be cross-reactive or polymorphic [33]. Indeed, focusing on the 99913 DVCs identified using iEVORA in the breast tissue study (Fig. 2b), we observed an overlap of 17206 CpGs with the 93382 cross-reactive and polymorphic probes of Chen et al. [33], representing an Odds Ratio (OR) overlap of 0.60, representing a significant underenrichment (one-tailed Fisher-test, *P* < 1e-50). Equivalently, by random chance, the overlap should have been around 19217 (Binomial test *P* < 1e-58). Restricting to the top 5000 hypervariable and hypermethylated DVCs of iEVORA, the overlap was 369 with an associated OR of 0.29, when by random chance the expected overlap should have been around 961, representing again a massive underenrichment (one-tailed Fisher test *P* < 1e-90, Binomial test *P* < 1e-100).

## Discussion

The data presented here strongly supports the view that DNA methylation alterations in pre-neoplastic cells are of an infrequent and hence stochastic nature, posing a statistical challenge to their identification. The epigenetic field defects were characterized by relatively few “outlier” samples exhibiting significant deviations in DNA methylation (at least 10 % changes in terms of absolute beta-values) from a normal ground state. Because of this, average levels in DNAm were not significantly changed, which is why ordinary t-tests or their non-parametric equivalents are underpowered to detect them. This problem is only exacerbated by the inherent difficulty to acquire sufficiently large numbers of normal tissue specimens from healthy and cancer patients. Thus, since increasing sample size is unrealistic, statistical methodologies which can increase the sensitivity of the assay offer the best hope to identify epigenetic field defects.

In light of this, the results presented here have deep and far-reaching implications: using two of the largest available DNA methylation data sets profiling precursor cancer lesions in two different cancer types, we have here shown that epigenetic field defects can only be identified if we adopt a feature selection paradigm based on differential variability. This substantially strengthens our previous observations [13, 14] and marks a paradigm shift for feature selection in the context of epigenetic field defect studies in cancer.

There are a number of other important observations that support, directly or indirectly, the statistical and biological significance of DVCs. First, we have seen that many of the DVCs defining epigenetic field defects progress to exhibit more frequent and therefore more homogeneous deviations in DNA methylation in samples that are neoplastic or invasive. Thus, the stochastic heterogeneity of DNAm deviations seen in pre-neoplastic lesions gives way to a much more homogeneous and deterministic pattern characteristic of neoplasia and cancer [15]. This is also the reason why ordinary t-tests, which largely assume homogeneous phenotypes, are perfectly adequate to identify cancer diagnostic markers [14]. Second, DVCs are not enriched for stromal or immune cell type GO-terms, instead exhibiting enrichment for binding sites of non cell-type specific TFs, strongly supporting the view that they do not reflect mean shifts in stromal or immune cell-type composition. Such mean shifts in stromal or immune cell-type composition, which could well be present in the tissues studied here, could be picked out by algorithms such as CAM or ISVA [34, 35], since these algorithms are designed to identify average shifts. However, given the complexity of epithelial tissue types [36, 37], we can’t discard that outlier DVCs may mark relatively large shifts in the epithelial cell subtype composition of the tissue. Indeed, the enrichment of DVCs in the matched breast cancers, may well reflect the proportional increase of the epithelial cell of origin of the tumour [22]. Third, and related to the previous point, we have demonstrated that DVCs exhibit a non-random genomic distribution, mapping preferentially to binding sites of key transcription factors specifying chromatin architecture. Fourth, DVCs identified using iEVORA are strongly underenriched for cross-reactive and polymorphic probes identified by Chen et al. [33]. This shows that such problematic probes are unlikely to give rise to DVCs, consistent with the view that DV between phenotypes is a biological feature. Finally, we have shown elsewhere that DVCs in normal breast tissue correlate with clinical features such as tumor size and clinical outcome [22].

Importantly, our statistical analysis also demonstrated that not all existing DV algorithms are able to identify epigenetic field defects. Indeed, we have seen that different DV algorithms exhibit widely different operating characteristics (especially power), largely dependent on the type of DV that is prominent within the carcinogenic stage under consideration. Thus, DV algorithms which can recognize heterogeneous DNA methylation outliers (such as our novel iEVORA algorithm), have the sensitivity to detect epigenetic field defects, whereas DV algorithms which only aim to control the type-1 error rate (J-DMDV & DiffVar) do not.

Of note, the better performance of iEVORA over the other DV algorithms was not just restricted to power, but also applied to the PPV. This is particularly noteworthy, because in theory, one main limitation of the Bartlett’s test implemented in iEVORA is the potentially large type-1 error rate. This explains why in some instances the PPVs were relatively low, e.g. as in the case of comparing normal to CIN2+ (PPV ~ 0.3), yet importantly the PPVs obtained from the other DV algorithms were generally even lower (Fig. 3a). We stress again that although the increased sensitivity afforded by iEVORA comes at the expense of a high FDR (or low PPV), that this is nevertheless preferable over using tests that yield zero sensitivity.

With regard to the PPV evaluation framework used in this manuscript, it is important to clarify a key and subtle point: the t-statistic computed in the independent data sets (representing more advanced cancer stages) to assess progression, has nothing to do with the t-statistic used in the iEVORA algorithm, which is only used to re-rank DVCs. It is important to realize that the use of a t-statistic to re-rank significant DVCs in the discovery set does not bias the PPV performance of iEVORA in the independent data sets. Indeed, we already previously showed that selecting and ranking features according to a t-test in the discovery set would yield worse PPVs in the independent data sets compared to the PPVs obtained using Bartlett’s test [14]. This indicates that CpGs exhibiting the most homogeneous changes (i.e. the largest absolute t-statistics) in the discovery set, are either not true positives (consistent with the large FDR values), or they reflect other biological effects which do not exhibit progression in more advanced cancer stages.

It is also worth emphasizing again the scenarios where we would expect iEVORA to be a useful feature selection tool. iEVORA is aimed at cancer studies where one is comparing two normal cellular phenotypes, with one of the phenotypes representing normal tissue at risk of neoplastic transformation. The two most common scenarios would include normal samples collected in a prospective setting, with a subset of the normal samples becoming cancerous at a later stage, or a comparison of normal tissue from healthy individuals to normal tissue found adjacent to the cancer. Such normal-adjacent tissue is “at risk” of neoplastic transformation, given that nearby tissue has already undergone transformation. We stress that iEVORA is not required and may even be counterproductive in scenarios where one wishes to identify diagnostic markers between normal and neoplasia or between normal and cancer tissue. The tissue being considered is also an important consideration, since our data only provides evidence for the biological importance of DNAm outliers in the actual tissue of origin for the cancer. Prospective or case–control cancer EWAS studies conducted in a surrogate tissue such as blood are scenarios where DNAm outliers are probably not of direct biological relevance to cancer development, and therefore represent situations where iEVORA is not appropriate, an observation we already made previously [14]. Indeed, we stress again that iEVORA has not been validated in general EWAS conducted in tissues such as blood, and therefore we advise the reader against its use in such studies, unless ample independent replication sets are available which would allow the biological and statistical significance of DVCs to be established. Even in the context of cancer field defect studies, given that the FDR and type-1 error rate can be high, application of a tool such as iEVORA is only advisable if independent data is available. This is a critical point, because DV can be driven by a whole plethora of factors, including genetic variation or exposure to unknown environmental factors. Only by testing DVCs in independent data, can one firmly establish their biological and statistical significance.

Finally, it is important to contrast the novel statistical methodology presented here to the feature selection method used in GWAS: there, one compares allelic frequencies between cases and controls. The direct analogue of this in EWAS is to search for loci that are altered as frequently as possible in cases compared to controls, i.e. to identify genomic sites where the mean level of DNAm differs as much as possible between the two phenotypes [38]. As we have seen however, such an approach is seriously underpowered in cancer studies where tissue availability is a major obstacle. The novel feature selection paradigm of DV offers a new dimension in the context of EWAS, where, in addition to allelic frequency, we also need to take the magnitude of the alteration into consideration. As shown here, infrequent but bigger changes in DNAm (thus defining outliers) are more likely to define cancer field defects, than more frequent yet smaller DNAm changes.

## Conclusions

In summary, we have here demonstrated that DNA methylation outliers in pre-neoplastic lesions define epigenetic field defects, marking cells which become enriched in invasive disease and which may therefore contribute casually to cancer progression. We recommend that studies aiming to identify epigenetic field defects in pre-neoplastic cells, and which for cost or logistical reasons may be underpowered, make use of DV algorithms like iEVORA, which improve the sensitivity, since this may be preferable over using algorithms which only provide strong control of the type-1 error rate and which therefore lack sensitivity.

## Ethical statement

All data analysed in this study is in the public domain and have been analysed in previous studies.

## Additional files

Additional file 1: Document containing all Supplementary Figures and Tables. (PDF 925 kb) Additional file 2: GSEA result tables of hypervariable DVCs, as identified using iEVORA, in the normal breast study comparing normal breast from healthy women to normal breast adjacent to breast cancer. There are 4 tables, corresponding to hypervariable DVCs mapping to TSS1500, TSS200 or 1^st^ Exon regions, and which are hypermethylated (dvUPdmUP) or hypomethylated (dvUPdmDN) in normal-adjacent tissue, as well as hypervariable DVCs mapping to gene-body or 5′UTR regions, which are hypermethylated (dvUPdmUP-GB) or hypomethylated (dvUPdmDN-GB) in normal-adjacent tissue. In each case, the columns label the number of genes in the MSigDB database list (nList), the number present prior to iEVORA analysis (nRep), the corresponding fraction (fRep), the number of genes overlapping with the iEVORA selected list (nOVLAP), the corresponding odds ratio (OR) and one-tailed Fisher test *P*-value (*P*-value), the adjusted *P*-value using Benjamini-Hochberg correction, and the gene symbols of the genes present in the overlap. (XLS 54 kb)

## Abbreviations

DMC: differentially methylated CpG
DNAm: DNA methylation
DV: differential variability
DVC: differentially variable CpG
EWAS: epigenome-wide-association study
FDR: false discovery rate
POI: phenotype of interest
