# Supplementary material for: Stochastic epigenetic outliers can define field defects in cancer
Source: BMC Bioinformatics. 2016 Apr 22;17:178. doi: 10.1186/s12859-016-1056-z (PMC4840974; doi:10.1186/s12859-016-1056-z)
Supplement: Additional file 1: — Document containing all Supplementary Figures and Tables. (PDF 925 kb) [file 12859_2016_1056_MOESM1_ESM.pdf]

Additional Data File 1

Stochastic epigenetic outliers can define field defects in cancer

Andrew E. Teschendorff [1,2,3,*] , Allison Jones [3] and Martin Widschwendter [3,*]

[*] Corresponding authors: Andrew E. Teschendorff- a.teschendorff@ucl.ac.uk, Martin Widschwendter- m.widschwendter@ucl.ac.uk

1. CAS Key Lab of Computational Biology, CAS-MPG Partner Institute for Computational Biology, Shanghai Institute for Biological Sciences, Chinese Academy of Sciences, Shanghai, China.
2. Statistical Cancer Genomics, Paul O'Gorman Building, UCL Cancer Institute, University College London, 72 Huntley Street, London WC1E 6BT, United Kingdom.
3. Department of Women's Cancer, University College London, 74 Huntley Street, London WC1E 6AU, United Kingdom.
4. University Clinic Erlangen, Department of Gynaecology and Obstetrics, Friedrich-Alexander University Erlangen-Nuremberg, Erlangen, Germany.

This SI document contains further details on Materials and Methods, all Supplementary Figures (S1-S5) and Supplementary Tables S1 & S2.

Supplementary Materials and Methods

We analysed a total of 5 DNA methylation data sets (**SI table S1**).

Precursor and cancer DNA methylation datasets: Our primary DNA methylation data sets focused on the profiling of precursor cancer lesions and are available from the GEO website (*www.ncbi.nlm.gov/geo*) under accession numbers GSE30758 and GSE69914.

Dataset GSE30758 consists of 152 cytologically normal cervical smear samples, representing prospectively collected samples within the ARTISTIC trial, with 75 of the women who provided a sample developing a cervical intraepithelial neoplasia of grade 2 or higher (CIN2+) three years after sample collection [1]. In order to test whether CpGs identified

from GSE30758, i.e. CpGs that correlate with the risk of CIN2+, show more progressive changes in CIN2+ and cervical cancer we used three other data sets (GSE20080, GSE37020, GSE30759) profiling normal cervical and CIN2+ or cervical cancer samples (**SI table S1**). All of these datasets were generated using Illumina Infinium 27k beadarrays and we used the normalized data, as described by us previously [1, 2].

Dataset GSE69914 was generated using Illumina Infinium 450k beadarrays and consists of 50 normal breast tissue samples from healthy women, a set of 42 matched normal-adjacent breast cancer pairs (a total of 84 samples), and a further 263 unmatched breast cancers. Raw data was processed (intra-sample normalization) with *minfi* [3] and type-2 probe bias corrected using BMIQ [4]. Subsequently, we tested for batch effects by performing a SVD on the intra-sample normalized data matrix, and checking which factors (biological or technical) the top components of variation were correlating with. The top components of variation in this data matrix correlated with biological factors, notably normal-cancer status.

Statistical algorithms for Differential Variability (DV)

We compared a total of 5 algorithms/statistical tests, aimed at identifying differentially variable features. The five DV algorithms/tests are (i) Bartlett's test [10], (ii) a novel DV algorithm, which we call "iEVORA" (similar to the original EVORA-Epigenetic Variable Outliers for Risk prediction Analysis algorithm [1, 2]), (iii) a joint test for differential means and differential variance in DNA methylation ("J-DMDV")[11], (iv) an empirical Bayes Levene-type test ("DiffVar") [12] and (v) a test based on a generalized additive model for location and scale ("GAMLSS") [13]. With the exception of iEVORA, which we present here for the first time, all other DV algorithms (i.e. BT/EVORA, J-DMDV, DiffVar, GAMLSS) have been previously used in cancer epigenome or EWAS studies [1, 13, 14].

BT & iEVORA: Briefly, Bartlett's test (BT) is similar to an F-test for testing homoscedasticity, and is well-known to be sensitive to single outliers. Because of this, we also consider a regularized version of it, which we call iEVORA, whereby features deemed significant by Bartlett's test are re-ranked according to an ordinary differential methylation statistic (e.g. the statistic from a t-test). Thus, in iEVORA, significance is assessed at the level of differential variability using Bartlett's test, but significant DV features with larger changes in the average DNA methylation are favored over those with smaller shifts in average DNA methylation. This re-ranking strategy therefore ensures that DV features driven by single, or a

few, outliers are only ranked highly if there are no features which are differentially methylated in terms of mean DNAm levels.

J-DMDV, DiffVar and *GAMLSS:* The third algorithm ("J-DMDV"), proposed by Wang and Ahn [11], works in the M-value ($M=\log_2[\beta/(1-\beta)]$ basis and uses a joint score test for mean and variance within a linear regression framework. The fourth algorithm ("DiffVar") is based on an empirical Bayes extension of the Levene-test [12]. Briefly, this algorithm first computes the square (or absolute) deviations of samples within a phenotype from the corresponding group (phenotype) mean using the M-value basis. It then uses the framework of moderated t-tests [15], to compare the distribution of deviations between the two phenotypes. The final algorithm ("GAMLSS") was developed by Wahl et al [13] within the GAMLSS (Generalized Additive Models for Location, Scale and Shape) framework. This algorithm also works in the M-value basis, and here we adapt it to run on 3 separate generalized linear additive models within a nested framework: a null model without mean and variance, a regression model for the mean only and a model for the mean and variance. Two likelihood ratio tests are then constructed by comparing the log-likelihoods of the mean-only model to the null, and the mean+variance model to the mean-only model. This yields two P-values for each feature, and features are deemed significant if at least one of these two P-values is less than a nominal threshold (after adjustment for multiple testing). Thus, GAMLSS will yield significant hits if there are differences in terms of mean DNAm. We also note that our implementation of GAMLSS does not compare a variance-only model to the null, since the algorithm aims to identify additional features where variance "adds predictive value" over a model which only includes the mean.

Evaluation of DV algorithms to detect true DVCs on simulated data

In order to compare the DV algorithms to each other, we devised a simulation framework allowing for different types of differential variability. In each simulation run we generated an artificial DNA methylation data matrix consisting of 6000 CpGs and 100 samples. Samples were subdivided into two phenotypes, a "normal" and a "disease state", each comprising 50 samples. We declared 600 CpGs to be truly differentially variable, allowing for 3 different types of DV, with 200 CpGs in each type. The remaining 5400 CpGs are not differentially variable. These are modelled from a beta-value distribution $B(a1,b1)$ with $a1=10$ and $b1=90$, i.e. we assume that these CpGs are generally unmethylated with a mean beta value of 0.1, with a standard deviation of approximately +/- 0.03. For the 600 true positives, a proportion

of the samples in the "disease" phenotype are modelled from a beta-value distribution *B(a2,b2)* with *a2=6* and *b2=4*, i.e. a distribution with mean value 0.6 and a standard deviation of approximately +/- 0.15. We note that although in this simulation we consider all CpGs to be unmethylated in the normal state, that there is no loss of generality, since mathematically, there is a complete symmetry between unmethylated and methylated CpGs. Thus, for the 600 true DVCs and for a number of samples in the disease phenotype, there will be an average increase in DNAm of ~0.5. The 600 true DVCs however fall into 3 categories of DV. For 200 of these CpGs, we model all samples in the disease phenotype from *B(a2,b2)*. Thus, these DVCs will typically also differ in terms of the mean level of DNA methylation and in fact, will exhibit stronger differences in terms of the mean DNAm than in terms of differential variance. Hence, these 200 DVCs are of "type-1a" DV. For another 200 CpGs, we only allow 20 of the 50 disease phenotype samples to be modelled from *B(a2,b2)*, with rest of the samples being modelled from *B(a1,b1)*. Thus, for these DVCs, half of the disease samples exhibit increases in DNAm, with the rest being indistinguishable from the normal phenotype. For these CpGs, differential variance is the key discriminatory characteristic, although they will still exhibit significant differences in terms of mean DNAm since a reasonable fraction of the disease samples exhibit deviations from the normal state. These DVCs are of "type-1b" differential variability. Finally, for the last set of 200 true positives, we only allow 3 disease samples to differ from the normal state. For these DVCs, there is therefore no significant difference in terms of the average DNA methylation between the two phenotypes. However, the variance will differ owing to the outliers in the disease phenotype. These DVCs are defined as being of "type-2".

We performed a total of 100 Monte Carlo runs, in each run recording 5 performance measures for each of the five DV algorithms: (1) the overall sensitivity of the DV algorithm using an FDR (false discover rate) corrected threshold of 0.05, (2) the true FDR at the estimated FDR < 0.05 threshold where the FDR estimate was obtained using Q-values [16], (3-5) the sensitivities to detect type-1a, type-1b and type-2 differentially variable CpGs. We focused on the FDR and not the FPR (false positive rate), since it is the FDR which gives us the confidence level that a given positive is a true positive, i.e. the FDR is related to the positive predictive value (PPV) through the relation FDR=1-PPV.

Evaluation of DV algorithms on real DNA methylation data

Initially, we compared the algorithms in their ability to detect DVCs between normal samples from healthy women and normal samples from women who developed neoplasia or who had cancer (see section on DNAm data sets for details), without considering the likelihood of these DVCs being true positives. Thus, for each of the DV algorithms and each CpG site, we estimated P-values, and from these, Q-values (FDR) [16]. In the case of BT and iEVORA, P-values came from Bartlett's test. In the case of DiffVar and J-DMDV, both tests provide P-values, as described in the respective publications. Features were deemed significant if Q<0.05. In the case of GAMLSS, we obtained two P-values, one assessing whether the mean is associated with the phenotype, and another assessing whether the variance adds predictive value over the mean. Both sets of P-values were transformed into Q-values and features with at least one of these Q-values being less than 0.05, were selected and deemed statistically significant.

To enable a more formal comparison of the DV algorithms, we devised a strategy that would allow us to estimate the positive predictive value (PPV) of the test. The key insight or hypothesis is that DVCs obtained from the previous step are more likely to be biological true positives if they exhibit progressive changes in DNA methylation in either neoplastic or invasive cancer tissue. A feature detected in pre-neoplastic lesions that is biological relevant is more likely to mark cells which become neoplastic and therefore one would expect enrichment of these marks in neoplasia and invasive cancer. This means that if a given "true positive" CpG site exhibits higher DNAm levels (maybe only marginally so) in precursor cancer lesions, that this same site will undergo larger and more frequent changes in DNAm when the cells are neoplastic or invasive. Statistically, this "progression effect" can be measured using t-statistics from a t-test, since the t-statistic is proportional to the average deviation in DNAm from the normal state. A similar argument can be applied to the case of CpG sites that undergo marginal hypomethylation in precursor cancer lesions.

In the context of cervical carcinogenesis, we thus applied the DV algorithms to identify DVCs hypervariable in the 75 normal samples which 3 years later progressed to CIN2+ status compared to the 77 normal samples from women remained healthy (the "ART" data set in **SI table S1**). The DNAm data for this set were generated on Illumina 27k beadarrays, and so, because of the design of the 27k array, we only focused on DVCs which exhibited increases in DNAm in the precursor lesions. We considered the top ranked 500, 1000 and 5000 DVCs (irrespective of FDR values attaining statistical significance). In the case of GAMLSS, which provides two P-values per feature, we ranked the selected features according to the significance of the DV statistic. In each case, we then computed and compared t-statistics of

these top ranked DVCs in two independent Illumina 27k data sets profiling normal and CIN2+ samples, and another 27k dataset of normal cervix and cervical cancers (“CIN2+(A)&(B)” and “CC” in **SI table S1**). The fraction of DVCs attaining t-statistics larger than 1.96 ($P<0.05$) and preserving the same directionality of change in the independent data was used as the PPV estimate.

In the context of breast carcinogenesis, we applied the DV algorithms to identify DVCs hypervariable in the 42 normal-adjacent samples compared to the 50 normal samples from healthy women. Because of the design of the 450k array, we now considered DVCs which exhibited either increases or decreases in DNAm in the normal-adjacent samples. We considered the top ranked 500, 1000 and 5000 DVCs in each category (irrespective of FDR values attaining statistical significance). In each case, we then computed t-statistics of these top ranked CpGs, as derived from comparing the 50 normal breast tissue samples to 305 breast cancers. The fraction of DVCs attaining t-statistics larger (lower) than 1.96 (-1.96) ($P<0.05$) and preserving the same directionality of change in the independent data, was used as the PPV estimate.

Supplementary Figures

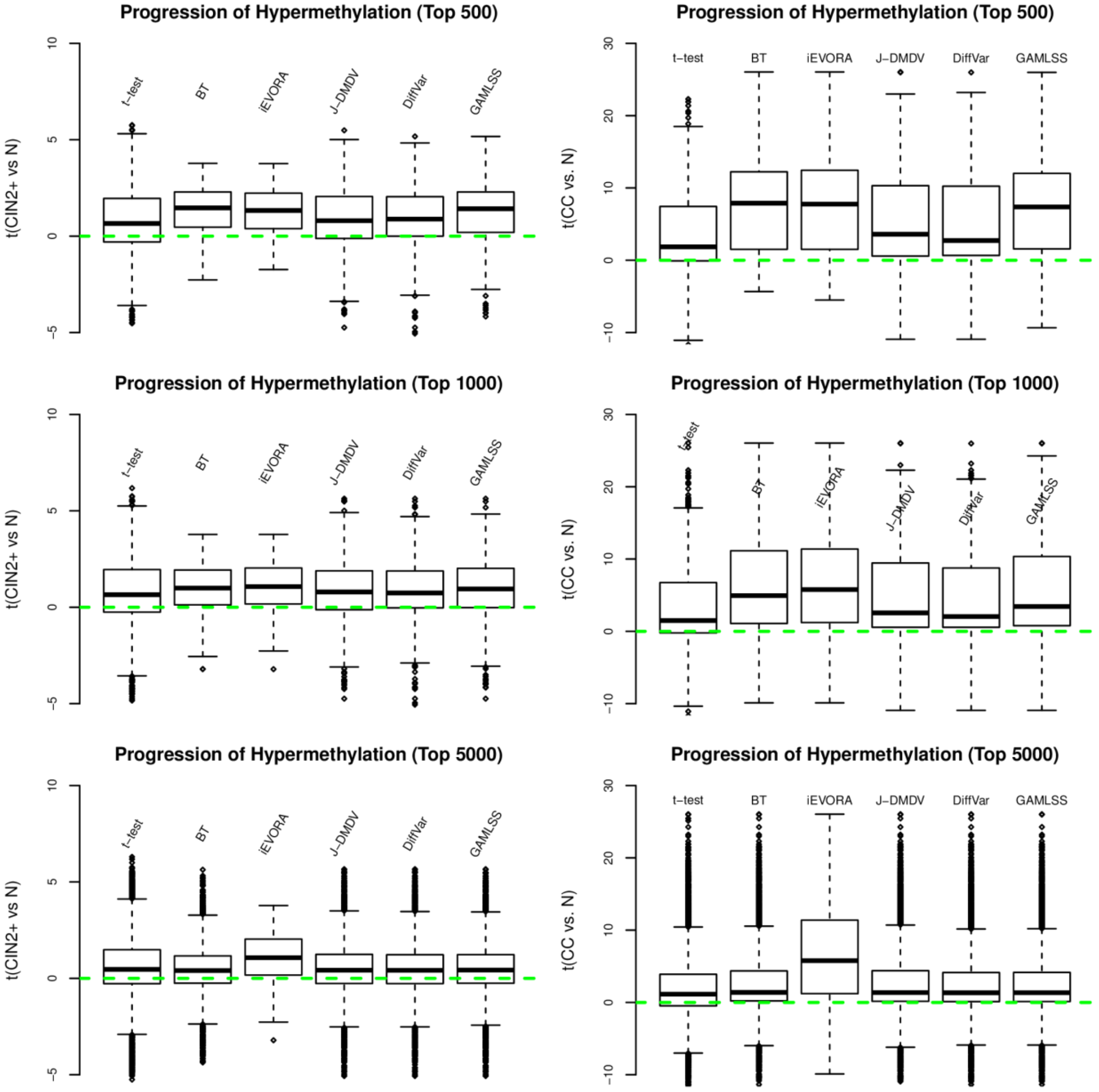

fig.S1: t-statistics of DMCs (t-tests) and DVCs (for DV algorithms), selected from the ARTISTIC cohort, in two independent data sets, one profiling 24 normal cervical smears (N) and 24 CIN2+ samples (left panels), and another profiling 15 normal cervical tissue (N) and 48 invasive cervical cancers (CC) (right panels). CpGs were selected from the ARTISTIC data set (comparing 75 normal cervical smear samples from women who 3 years later developed a CIN2+ to 77 from women who remained disease free) according to one of the 6 feature selection algorithms. Because of the design of the 27k beadarray, only CpGs hypermethylated in the prospective CIN2+ samples were considered. Top panels are for the top ranked 500 CpGs, middle panels for the top rank 1000 CpGs, lower panels for the top ranked 5000. In the panels, those CpGs exhibiting progression should have positive t-

statistics, and thus we see that CpGs selected and ranked using iEVORA generally do show more consistent progressive changes.

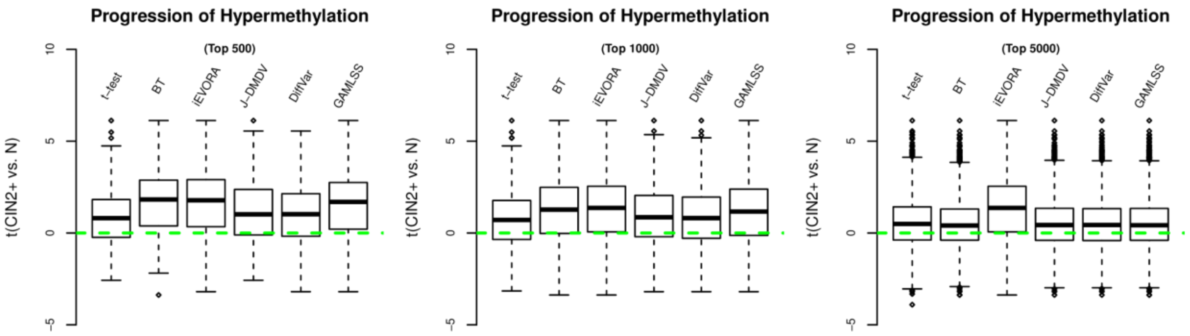

fig.S2: t-statistics of DMCs (t-tests) and DVCs (for DV algorithms), selected from the ARTISTIC cohort, in another independent data set, profiling 30 normal cervical smears (N) and 18 CIN2+ samples. Further details as in fig.S3.

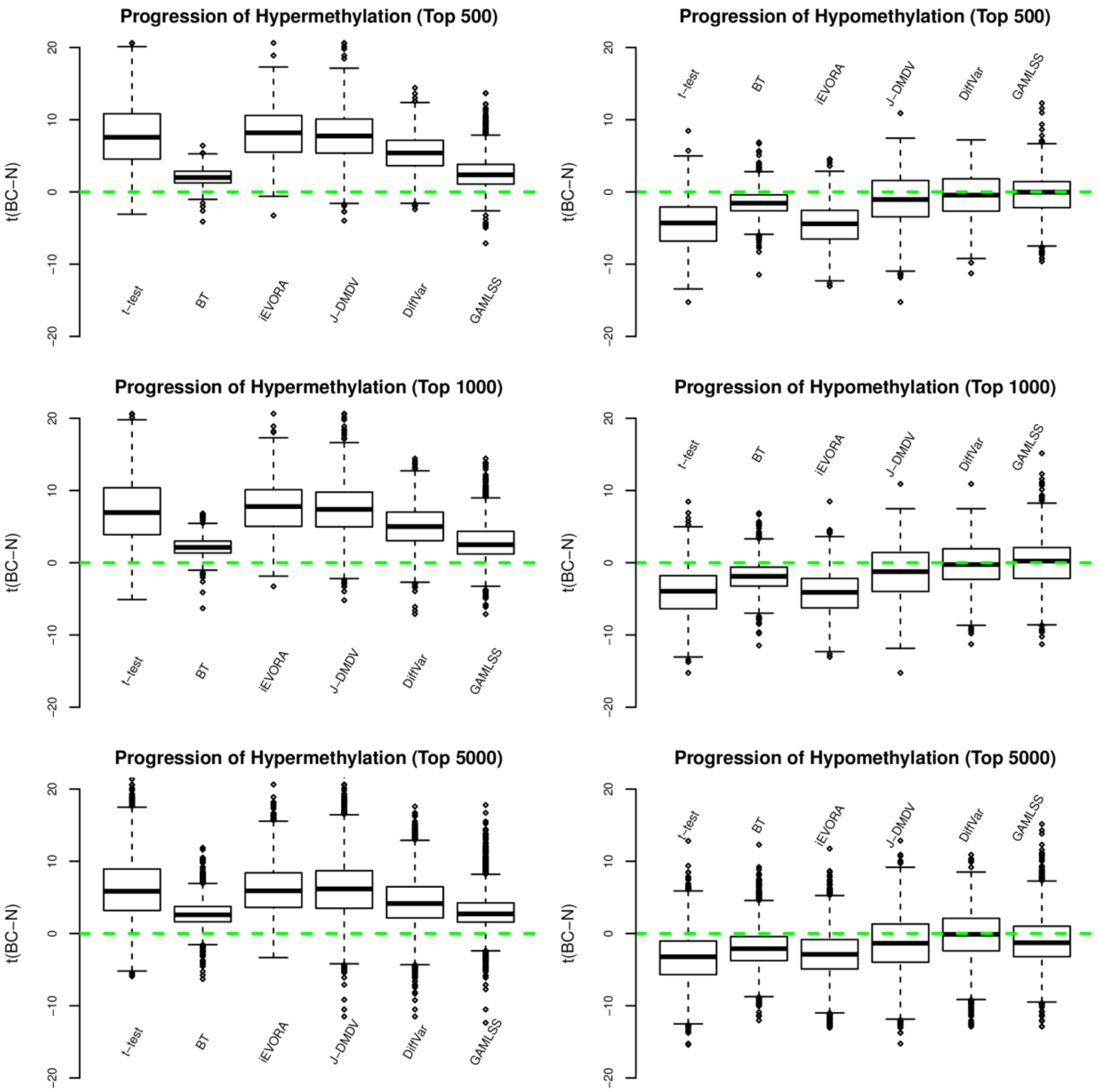

fig.S3: t-statistics of CpGs, selected using one of the 6 feature selection algorithms from comparing 50 normal breast samples (N) to 42 normal samples collected adjacent (NADJ) to breast cancers, with the t-statistics computed by comparing the 50 normal breast samples to 305 breast cancers (BC). Hypermethylated and hypomethylated CpGs were ranked separately, using one of the 6 different algorithms, as indicated. Panels are for the top ranked 500, 1000 and 5000 CpGs. In the left panels, those CpGs exhibiting progressive hypermethylation should have positive t-statistics, whereas those in the right panels showing progressive hypomethylation should have negative t-statistics, and thus we see that DVCs selected and ranked using iEVORA generally show the most consistent progressive changes.

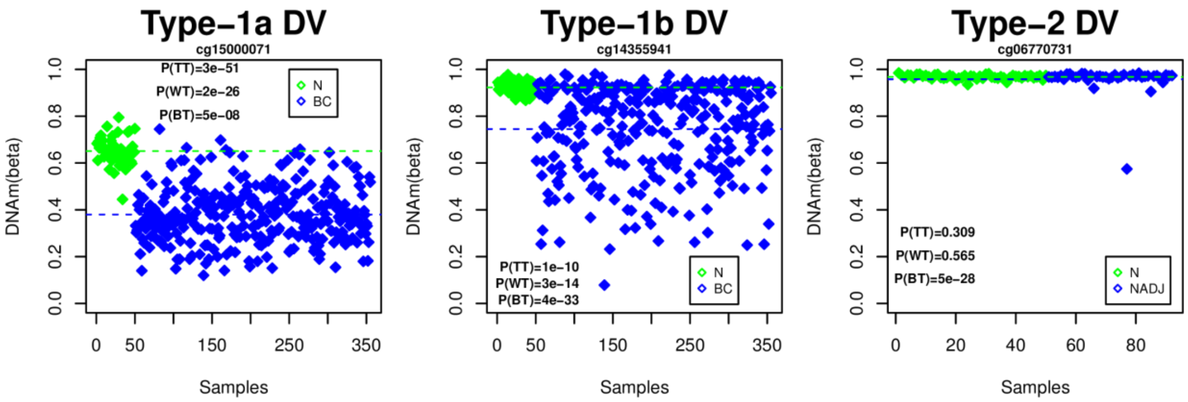

fig.S4: Real data examples of different types of DV in breast carcinogenesis involving hypomethylation in the disease state. The horizontal dashed lines indicate the mean in each phenotype. Phenotype is labelled by a different color. N=normal, NADJ=normal breast tissue adjacent to a cancer, BC=breast cancer. P-values from a t-test (TT), a Wilcoxon rank sum test (WT) and Bartlett's test (BT) are given. Bartlett's test is a test for differential variance.

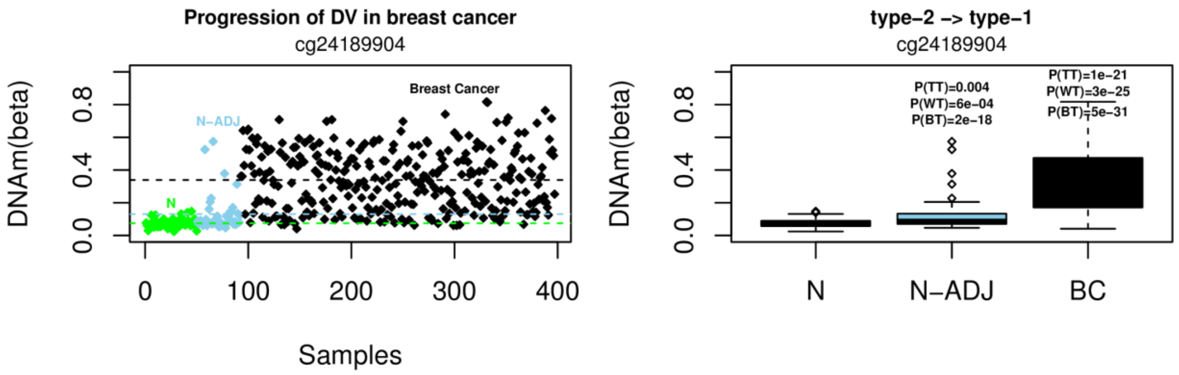

fig.S5: Progression of DV in breast carcinogenesis. Left panel depicts the DNAm fraction of a specific CpG (cg24189904) across 3 different disease stages in breast carcinogenesis, including normal cells (N), normal cells adjacent to a breast cancer (N-ADJ) and breast cancer (BC). **Right panel** is a boxplot representation, indicating the P-values from a t-test (TT), Wilcoxon rank sum test (WT) and Bartlett's test (BT) between the normal state and each of N-ADJ and BC.

Supplementary Tables

Dataset	Total number of samples	Tissue type	Phenotypes	Accession
ART	152	Cervix	75 preCIN2+ and 77 normal	GSE30758
CIN2+(A)	48	Cervix	24 normal and 24 CIN2+ (all HPV+)	GSE37020
CIN2+(B)	48	Cervix	30 normal + 18 CIN2+	GSE20080
CC	63	Cervix	15 normal + 48 cervical cancers	GSE30759
PREBC	397	Breast	50 normals + 42 matched normal-tumor pairs (84) + 263 unmatched tumors	GSE69914

table S1- Datasets used in this study. Table lists details of the 5 DNA methylation datasets considered in this work. We provide the name of the cohort/study, the total of number of samples used, the tissue/cell-type, the distribution of samples according to the various phenotypes and finally the data accession number.

TF ChIP-Seq experiment	OR(dvUPdmUP)	P(dvUPdmUP)	OR(dvUPdmDN)	P(dvUPdmDN)
EZH2_39875_None__Broad	6.22	< 1E-100	0.03	1
RBBP5_A300.109A_None__Broad	2.06	9.00E-54	0.01	1
SUZ12_None__USC	4.89	4.00E-44	0.26	0.996
CTCF_None__Broad	2.29	2.00E-33	0.33	1

Rad21_None__Stanford	2.49	2.00E-33	0.17	1
CTCF_None__UT.A	2.61	3.00E-33	0.27	1
CtBP2_None__USC	2.37	2.00E-32	0.03	1
Rad21_None__HudsonAlpha	2.24	5.00E-32	0.27	1
CTCF_SC.5916_None__HudsonAlpha	2.36	2.00E-28	0.24	1
Znf143_16618.1.AP_None__Stanford	1.94	5.00E-24	0.1	1
NRSF_None__HudsonAlpha	2.47	5.00E-14	0.09	1
SIN3A_NB600.1263_None__Stanford	1.45	2.00E-13	0.01	1
CHD1_A301.218A_None__Broad	1.84	3.00E-13	0.07	1
TBP_None__Stanford	1.48	8.00E-13	0.02	1
Max_None__USC	1.72	6.00E-11	0.03	1
GABP_None__HudsonAlpha	1.77	6.00E-10	0.04	1
TAF1_None__HudsonAlpha	1.36	7.00E-10	0.01	1
Bach1_sc.14700_None__Stanford	1.67	2.00E-09	0.03	1
Egr.1_None__HudsonAlpha	1.65	3.00E-09	0.03	1
TEAD4_SC.101184_None__HudsonAlpha	1.75	2.00E-08	0.1	1
HDAC2_SC.6296_None__HudsonAlpha	1.76	1.00E-05	0	1
YY1_SC.281_None__HudsonAlpha	1.35	3.00E-05	0.09	1
TAF7_SC.101167_None__HudsonAlpha	1.33	5.00E-05	0.02	1
USF.1_None__HudsonAlpha	1.41	1.00E-04	0.07	1
Mxi1_AF4185_None__Stanford	1.36	4.00E-04	0	1
USF2_None__Stanford	1.45	0.001	0.06	1
Sin3Ak.20_None__HudsonAlpha	1.3	0.001	0	1
ATF2_SC.81188_None__HudsonAlpha	1.33	0.002	0.04	1
JunD_None__Stanford	1.37	0.002	0	1
SP4_V.20_None__HudsonAlpha	1.24	0.002	0	1
MafK_ab50322_None__Stanford	1.72	0.003	0.18	0.996
TCF12_None__HudsonAlpha	1.45	0.006	0.18	1
JunD_None__HudsonAlpha	1.31	0.008	0	1
p300_None__HudsonAlpha	1.28	0.009	0.04	1
SP1_None__HudsonAlpha	1.19	0.013	0	1
c.Myc_None__UT.A	2.28	0.015	1.61	0.353
Pol2_None__HudsonAlpha	1.11	0.025	0.02	1
GTF2F1_AB28179_None__Stanford	1.21	0.036	0	1
NANOG_SC.33759_None__HudsonAlpha	1.41	0.059	0	1
CHD1_A301.218A_None__Stanford	1.27	0.073	0.2	0.999
RFX5_200.401.194_None__Stanford	1.36	0.082	0	1
Nrf1_None__Stanford	1.17	0.094	0	1
CHD2_AB68301_None__Stanford	1.12	0.109	0	1
Pol2.4H8_None__HudsonAlpha	1.08	0.112	0.04	1
SRF_None__HudsonAlpha	1.18	0.152	0	1
c.Jun_None__Stanford	1.31	0.16	0	1
c.Myc_None__Stanford	1.13	0.17	0	1
BCL11A_None__HudsonAlpha	1.69	0.179	0	1
FOSL1_SC.183_None__HudsonAlpha	1.24	0.262	0.34	0.948
RXRA_None__HudsonAlpha	1.14	0.407	0	1

Pol2_None__UT.A	1.02	0.408	0.01	1
ATF3_None__HudsonAlpha	0.99	0.552	0	1
SP2_SC.643_None__HudsonAlpha	0.95	0.658	0.06	1
CEBPB_None__Stanford	0.93	0.686	0	1
POU5F1_SC.9081_None__HudsonAlpha	0.8	0.759	0.95	0.621
SIX5_None__HudsonAlpha	0.9	0.772	0	1
BRCA1_A300.000A_None__Stanford	0.81	0.894	0	1
JARID1A_ab26049_None__Broad	0.4	1	0	1

table S2: Odds Ratio (OR) and one-tailed Fisher-test P-values of enrichment of 58 TF ChIP-Seq binding sites, as determined in hESCs, among two classes of DVCs identified using iEVORA comparing normal breast tissue from healthy women to normal tissue adjacent to breast cancers: hypervariable + hypermethylated (dvUPdmUP) and hypervariable + hypomethylated (dvUPdmDN).

References

1. Teschendorff AE, Jones A, Fiegl H, Sargent A, Zhuang JJ, Kitchener HC, Widschwendter M: **Epigenetic variability in cells of normal cytology is associated with the risk of future morphological transformation.** *Genome Med* 2012, **4:**24.
2. Teschendorff AE, Widschwendter M: **Differential variability improves the identification of cancer risk markers in DNA methylation studies profiling precursor cancer lesions.** *Bioinformatics* 2012, **28:**1487-1494.
3. Aryee MJ, Jaffe AE, Corrada-Bravo H, Ladd-Acosta C, Feinberg AP, Hansen KD, Irizarry RA: **Minfi: a flexible and comprehensive Bioconductor package for the analysis of Infinium DNA methylation microarrays.** *Bioinformatics* 2014, **30:**1363-1369.
4. Teschendorff AE, Marabita F, Lechner M, Bartlett T, Tegner J, Gomez-Cabrero D, Beck S: **A beta-mixture quantile normalization method for correcting probe design bias in Illumina Infinium 450 k DNA methylation data.** *Bioinformatics* 2013, **29:**189-196.
5. Paul D: **An epigenome-wide association study for type-1 diabetes.** 2015.
6. Nordlund J, Bäcklin CL, Wahlberg P, Busche S, Berglund EC, Eloranta M-L, Flaegstad T, Forestier E, Frost B-M, Harila-Saari A, et al: **Genome-wide signatures of differential DNA methylation in pediatric acute lymphoblastic leukemia.** *Genome Biol* 2013, **14:**R105.
7. Johnson WE, Li C: **Adjusting batch effects in microarray expression data using empirical Bayes methods.** *Biostatistics* 2007, **8:**118-127.
8. Leek JT, Johnson WE, Parker HS, Jaffe AE, Storey JD: **The sva package for removing batch effects and other unwanted variation in high-throughput experiments.** *Bioinformatics* 2012, **28:**882-883.
9. Rakyan VK, Beyan H, Down TA, Hawa MI, Maslau S, Aden D, Daunay A, Busato F, Mein CA, Manfras B, et al: **Identification of type 1 diabetes-associated DNA methylation variable positions that precede disease diagnosis.** *PLoS Genet* 2011, **7:**e1002300.
10. Snedecor GW, Cochran WG: *Statistical Methods.* 1989.

11. Ahn S, Wang T: **A powerful statistical method for identifying differentially methylated markers in complex diseases.** *Pac Symp Biocomput* 2013**:**69-79.
12. Phipson B, Oshlack A: **DiffVar: a new method for detecting differential variability with application to methylation in cancer and aging.** *Genome Biol* 2014, **15:**465.
13. Wahl S, Fenske N, Zeilinger S, Suhre K, Gieger C, Waldenberger M, Grallert H, Schmid M: **On the potential of models for location and scale for genome-wide DNA methylation data.** *BMC Bioinformatics* 2014, **15:**232.
14. Xu X, Su S, Barnes VA, De Miguel C, Pollock J, Ownby D, Shi H, Zhu H, Snieder H, Wang X: **A genome-wide methylation study on obesity: Differential variability and differential methylation.** *Epigenetics* 2013, **8**.
15. Smyth GK: **Linear models and empirical bayes methods for assessing differential expression in microarray experiments.** *Stat Appl Genet Mol Biol* 2004, **3:**Article3.
16. Storey JD, Tibshirani R: **Statistical significance for genomewide studies.** *Proc Natl Acad Sci U S A* 2003, **100:**9440-9445.
